# Drug resistance in HIV patients with virological failure or slow virological response to antiretroviral therapy in Ethiopia

**DOI:** 10.1186/1471-2334-14-181

**Published:** 2014-04-04

**Authors:** Alemseged Abdissa, Daniel Yilma, Jannik Fonager, Anne M Audelin, Lone H Christensen, Mette F Olsen, Markos Tesfaye, Pernille Kaestel, Tsinuel Girma, Abraham Aseffa, Henrik Friis, Court Pedersen, Aase B Andersen

**Affiliations:** 1 Department of Medical Laboratory Sciences & Pathology, Jimma University, Jimma, Ethiopia; 2 Department of Infectious Diseases, Odense University Hospital, Odense, Denmark; 3 Department of Internal Medicine, Jimma University, Jimma, Ethiopia; 4 Department of Microbiological Diagnostics & Virology, Statens Serum Institut, Copenhagen, Denmark; 5 Department of Nutrition, Exercise and Sports, University of Copenhagen, Copenhagen, Denmark; 6 Department of Psychiatry, Jimma University, Jimma, Ethiopia; 7 Department of Pediatrics and Child Health, Jimma University, Jimma, Ethiopia; 8 Armauer Hansen Research Institute, Addis Ababa, Ethiopia

## Abstract

**Background:**

The ongoing scale-up of antiretroviral therapy (ART) in sub-Saharan Africa has prompted the interest in surveillance of transmitted and acquired HIV drug resistance. Resistance data on virological failure and mutations in HIV infected populations initiating treatment in sub-Saharan Africa is sparse.

**Methods:**

HIV viral load (VL) and resistance mutations pre-ART and after 6 months were determined in a prospective cohort study of ART-naïve HIV patients initiating first-line therapy in Jimma, Ethiopia. VL measurements were done at baseline and after 3 and 6 months. Genotypic HIV drug resistance (HIVDR) was performed on patients exhibiting virological failure (>1000 copies/mL at 6 months) or slow virological response (>5000 copies/mL at 3 months and <1000 copies/mL at 6 months).

**Results:**

Two hundred sixty five patients had VL data available at baseline and at 6 months. Virological failure was observed among 14 (5.3%) participants out of 265 patients. Twelve samples were genotyped and six had HIV drug resistance (HIVDR) mutations at baseline. Among virological failures, 9/11 (81.8%) harbored one or more HIVDR mutations at 6 months. The most frequent mutations were K103N and M184VI.

**Conclusions:**

Our data confirm that the currently recommended first-line ART regimen is efficient in the vast majority of individuals initiating therapy in Jimma, Ethiopia eight years after the introduction of ART. However, the documented occurrence of transmitted resistance and accumulation of acquired HIVDR mutations among failing patients justify increased vigilance by improving the availability and systematic use of VL testing to monitor ART response, and underlines the need for rapid, inexpensive tests to identify the most common drug resistance mutations.

## Background

In Ethiopia approximately 1.3 million people live with HIV and the estimated adult HIV prevalence is 1.5% [1]. During the past eight years, there has been a rapid scale-up of antiretroviral therapy (ART) which reached 250,000 adults in 2012, representing 86% of eligible patients [2,3].

As ART roll-out continues in resource-limited settings, the risk of potential emergence of HIV drug resistance (HIVDR) is growing. This could be due to the absence of virological monitoring in routine clinical care and the use of drugs with low genetic barriers such as non-nucleoside reverse transcriptase inhibitors [NNRTI] [4]. The use of nevirapine as monotherapy for preventing mother-to-child transmission may have further contributed to the problem [5].

Viral replication under suboptimal antiretroviral pressure leads to accumulation of resistance mutations, which limit future therapeutic choices [6] as mutations conferring resistance to one drug frequently confer cross resistance to other antiretroviral drugs within the same class [7]. Thus, it is essential for patient management to define the pattern of both primary and secondary resistance mutations.

The World Health Organization (WHO) recommends surveillance for transmitted HIVDR among antiretroviral-naïve patients and drug resistance mutations emerging during treatment in all countries involved in the ARV access programs [8,9]. Although transmitted and acquired HIV-1 drug resistance mutations have been well-described and longitudinally surveyed in high-income countries such as France [10], United States [11] and Denmark [12] there are few data on the subject in resource-limited settings.

The aim of our study was to assess the prevalence of virological failure and resistance mutations in patients initiating treatment in Ethiopia.

## Methods

### Study setting

The present study was part of a randomized controlled trial evaluating two nutritional supplements in adult patients initiating ART in Ethiopia, which was registered at http://www.controlled-trials.com (ISRCTN32453477). There were no differences in virological suppression among intervention groups (Olsen MF et al., submitted). The Ethiopian ART program was initiated in 2005 free of charge in hospitals and followed by the expansion to health centers in the periphery in 2007. Patients initiating ART at Jimma University Hospital, Jimma Health Center and Agaro Health Center were enrolled between July 2010 and August 2012. The first two centers are located in Jimma (a city of 150,000 inhabitants), with an urban site managing a total of approximately 1,617 patients on ART in Jimma University Hospital and 317 in Jimma Health center. The third centre is located in Agaro (a small town of 28,000 inhabitants) with approximately 240 patients on treatment.

### Study participants

HIV-positive patients attending the ART clinics, who were ART naïve and eligible for ART according to the Ethiopian national guideline [13], were invited to participate in the study and followed for 6 months. Inclusion criteria were being adult (≥18 years), living within approximately 50 km of Jimma, and consenting to participate. Pregnant or lactating women and patients with known previous use of ART were excluded. Participants gave informed consent and protocols were approved by Jimma University Ethics Review Board and National Research Ethics Committee of Ethiopia.

Decision to initiate treatment was made according to WHO criteria: stage IV irrespective of CD4 count, stage III if CD4 < 350, or CD4 < 200 cells/μl at any stage. The choice of ART combination was guided by national treatment guideline [13] and generally consisted of one of three first line regimens [Tenofovir (TDF) + Lamivudine (3TC) + Nevirapine (NVP)/Efavirenz (EFV); or Zidovudine (AZT) + 3TC + NVP/EFV; or Stavudine (d4T) + 3TC + NVP/EFV]. As part of the ART program, patients collected drugs every month for free. CD4 counts were monitored at ART initiation and every 6 months. Viral load monitoring was performed as part of this study.

### Specimen collection and processing

Whole blood was obtained for CD4 count and plasma samples were separated and stored at −80°C until analyzed for HIV viral load and genotype. CD4 count and viral loads were measured at baseline and after 3 and 6 months on therapy. Genotypic HIVDR was done at baseline and after 6 months among selected participants based on viral load levels.

### Viral load and CD4 count

HIV-1 viral load (VL) was quantified using a commercial PCR assay (RealTime HIV-1, Abbott Laboratories, Illinois, USA) and automated extraction system (m2000 Real Time System, Abbott Laboratories, Illinois, USA) was used. Extracted samples were amplified and detected on the *m2000rt* platform (Abbott Laboratories, Illinois, USA). Virological failure was defined as a confirmed VL >1000 copies/mL at 6 months and viral suppression is the success of attaining VL <1000 copies/mL at 6 months. Virological slow responders were defined as VL ≥5000 copies/mL at 3 months but with viral suppression at 6 months. CD4 cells were enumerated using the Facscount® automated cell counter (Becton-Dickinson, Franklin Lakes, New Jersey, USA). Immunological failure was defined as a decline in CD4 count from baseline after 6 months of ART [14].

### Adherence to the ART

Self-reported adherence to ART was documented monthly. In addition, efavirenz or nevirapine plasma concentrations were measured at 1 and 2 months using liquid chromatography-tandem mass spectrometry at Division of Clinical Pharmacology, university of Cape Town, South Africa. Limit of quantification was 0.01 μg/ml for both drugs.

### Resistance genotyping

Plasma samples were shipped to Copenhagen, Denmark on dry ice for drug resistance mutation analysis at Statens Serum Institut (SSI, Copenhagen DK). Amplification of the pol gene was performed in two steps using SuperScript™ III One-Step RT-PCR System with Platinum® Taq High Fidelity (Invitrogen) in accordance with manufacturer´s instructions. First cDNA synthesis and first PCR was performed using the primers JA203 [15] and IN3 [16] at the following conditions: 52°C 30 min, 94°C, 2 min followed by 40 cycles of: 94°C, 15 sec; 60°C, 45 sec; 66°C, 3 min and a final extension at: 66°C for 10 min. The second nested-PCR was performed using the Expand High Fidelity PCR System (Roche) in accordance with manufacturer´s instructions and with the primers JA204 [15] and ABI1_R [17] at the following conditions: 94°C, 2 min followed by 35 cycles of: 94°C, 30 sec; 60°C, 30 sec; 72°C, 60 sec, and a final extension at: 72°C for 7 min, yielding a PCR fragment of 1449 bp. PCR products were prepared for sequencing using Illustra^TM^ ExoProStar 1 step (GE Healthcare) and sequencing reactions were carried out using the BigDye Terminator version 1.1 (Applied Biosystems) and the sequencing primers:

INH-A:CTTCAGAGCAGACCAGAGCCAA,

INH-B:GTTAAACAATGGCCATTGACAGA,

INH-D:CAGRCYGGAGCCAACAGC,INH-C:GATGGAAAGGATCACCRGCAATA, INH-F:TTGGGCCATCCATTCCTGG,

INH-G:CCATCCCTGTGGAAGCACAT

INH-H:TTCTGTATTTCTGCTAYTAAGTCTTTTG.

Sequencing was performed on an ABI 377 DNA Sequencer (Applied Biosystems) and sequence assembly and analysis was performed in BioNumerics v. 6.6 (Applied Maths, Sint-Martens-Latem, Belgium). For six of the 29 analyzed samples (Id #_month; 25_6, 146_6, 301_6, 314_6, 360_6 and 25_0), sequences were obtained using the ViroSeq HIV-1 genotyping System v. 2 (Abbott Diagnostics, Foster City, CA) in accordance with manufacturer´s instructions.

### Drug resistance mutation analysis and statistical methods

For each sequence, the resistance profile was calculated using the current HIVdb algorithm [18] at Stanford’s HIV genotypic resistance profile (http://sierra2.stanford.edu/sierra/servlet/JSierra?action=sequenceInput) and as implemented in BioNumerics v. 6.6.

Statistical analysis was done using STATA/IC version 11.2 (StataCorp LP, College Station, USA). Differences in means, medians and proportions between men and women were tested using Pearson Chi-square test and the Fisher’s exact test. P-values <0.05 were considered significant.

### Sequences submitted to GenBank

Sequences obtained in this study were submitted to GenBank under the following accession numbers: KJ561122-KJ561136 (baseline samples) and KJ561137-KJ561150 (6 months samples).

## Results

### Participants and their baseline characteristics

A total of 312 adult patients on first-line ART were enrolled into the study. Of these, 281 and 273 had reached 3 and 6 months of follow up, respectively, while the rest dropped out for various reasons including withdrawal of consent, lost to follow up or death. A total of 275 participants had VL results available at 3 months and 265 at 6 months. At ART initiation, the 265 participants retained for 6 months of follow-up did not differ significantly from the rest of participants (n =47), in terms of baseline characteristics such as age, BMI, VL and WHO stage. The mean (±SD) age was 33.0 (±8.8) years and 67% were women (Table 1). A combination of tenofovir (TDF), lamuvidine (3TC) and efavirenz (EFV) or nevirapine (NVP) was the most commonly (82.6%) prescribed first-line regimen. The other first-line regimens contained zidovudine (AZT; 14.3%) or stavudine (d4T; 3%) instead of TDF.

**Table 1:** Baseline characteristics of HIV-1 positive participants treated with first-line ART regiments in Jimma, Ethiopia (n =312)

	**Women (n =179)**	**Men (n =86)**	**Total (n = 265)**	**Excluded, did not complete 6 months (n =47)**
Age, y	31.0 ±8.0	38.0 ±9.0	33.0 ±8.8	32.2
Body mass index (BMI), kg/m^2^	19.6 ±2.5	19.1 ±1.8	19.4 ±2.3	19.5 ±2.5
CD4 count at enrollment, cells/ul	189 ±113	194 ±113	190.7 ±113	181 ±105
Viral load, log(copies/mL )	4.7 ±1.0	4.6 ±0.9	4.7 ±1.0	4.8 ±0.9
WHO stage				
Stage I	63 (35.2)	23 (26.7)	86 (32.5)	11 (23.4)
Stage II	47 (26.3)	32 (37.2)	79 (29.8)	12 (25.5)
Stage III	53 (29.6)	26 (30.2)	79 (29.8)	19 (40.4)
Stage IV	16 (8.9)	5 (5.8 )	21 (7.9)	5 (10.6)
ART regimen				
TDF/3TC/EFV	113 (63.1)	77 (89.5)	190 (71.7)	33 (70.2)
AZT/3TC/EFV	2 (1.1)	1 (1.2)	3 (1.1)	1 (2.1)
TDF/3TC/NVP	27 (15.1)	2 (2.3)	29 (10.9)	3 (5.7)
AZT/3TC/NVP	31 (17.3)	4 (4.7)	35 (13.2)	10 (18.9)
Other*	6 (3.4)	2 (2.3)	8 (3.0)	0 (0.0)

*Other regimen includes mainly D4T and NVP based treatment. Data are mean ±SD or n (%).

### Virological outcomes and immunological criteria

Virological failure was observed in 14 (5.3%) of the participants. Three of the participants with good viral suppression at 6 months had a slow response. These participants (Id # 146, 301 and 314) had 184878, 6849 and 125562 copies/mL at 3 months, respectively (Table 2). It was found that 158/275 (57.5%) and 233/265 (87.9%) of the participants achieved VL <40 copies/mL at 3 and 6 months respectively. Patients experiencing virological failure had significantly lower CD4 count at 6 months mean (±SD) =169 (±85) compared to those with successful viral suppression 313 (±131); P = 0.002). However, only four of the 14 participants with virological failure (28.6%) experienced immunological failure at 6 month (Table 2) and, 22 of 251 (8.7%) participants with virological suppression experienced immunological failure.

**Table 2:** Characteristics of participants with virological failure and slow virological responders

			**Viral load (copies/mL)**		**CD4 (cells/μl)**					
**Patient Id**	**Age**	**Sex**	**At 3 months**	**At 6 months**	**At baseline**	**At 6 months**	**Decline in CD4 count**	**NNRTI conc, μg/ml at 1 month**	**NNRTI conc, μg/ml at 2 months**	**>95% reported adherence**
**Virological failures**										
**9**	40	M	16816	7703	124	235	No	2.16	2.27	Yes
**25**	39	M	54	116373	114	163	No	4.6	4.64	Yes
**157**	47	M	91377	51498	41	153	No	2.9	Missing	Yes
**164**	22	F	<40	11420	127	178	No	3.88	1.39	Yes
**213**	35	F	767340	29427	114	251	No	<0.01	<0.01	Yes
**243**	26	F	<40	163259	207	116	Yes	8.7	8.04	Yes
**269**	30	F	86463	66498	207	331	No	<0.01	0.01	Yes
**300**	50	F	Undetectable	28038	119	199	No	Missing	2.48	Yes
**339**	30	F	42735	381889	19	81	No	10.9	10.1	Yes
**346**	25	F	Missing	2002	225	357	No	5.34	Missing	Yes
**354**	40	M	12237	30252	137	122	Yes	2.32	2.94	Yes
**357**	30	F	24931	143175	221	240	No	<0.01	<0.01	Yes
**360**	32	F	7289	111647	287	192	Yes	18.6	Missing	Yes
**373**	34	F	344294	78504	48	5	Yes	<0.01	<0.01	Yes
**Slow virological responders**										
**146**	20	F	184878	141	131	360	No	4.42	6.54	Yes
**301**	30	F	6849	640	79	214	No	4.33	Missing	No
**314**	50	F	125562	379	72	72	No	Missing	<0.01	No

### HIVDR mutations at baseline

HIVDR genotyping was performed in pre-ART plasma specimens from 12 participants with virological failure. All patients were infected with HIV subtype C. Resistances to NNRTIs were identified in six of 12 (50%) and none were resistant to NRTI. Mutations detected in three of the patients are associated with high level resistance. Five participants with HIVDR mutations at ART initiation received ART regimens that were only partially active (Additional file 1: Table S1). During the 6 months of follow-up, none of these patients were switched to a fully active first-line or second-line regimen.

A total of seven patients had HIVDR mutations detectable in the baseline samples; one mutation (V90IV) confers no resistance (Id # 339). The K103N/X was the most common mutation observed. However, one participant (Id # 25) had a mutation (A98G) that confers resistance to nevirapine. This patient was treated with a supposedly active efavirenz based regimen and the mutation had disappeared at 6 months (Additional file 1: Table S1).

### HIVDR mutations at 6 months

HIVDR genotyping was performed in plasma from 11 participants with virological failure at 6 months. Nine of them (81.8%) harboured mutations that cause high level resistance to NNRTIs. Six participants had the mutations at baseline, and three (Id # 9, 339 and 346) acquired additional mutations during follow-up causing resistance to NRTI. Three of the participants with HIVDR had no resistance at baseline but acquired resistance mutations during the course of treatment and two of them became resistant to both NNRTIs and NRTIs. HIVDR genotyping could not be performed for 3 participants (Id # 164, 354 and 373) due to failed sequencing attempt or sample inadequacy (Additional file 1: Table S1).

Of the three slow responders, one (Id # 301) was resistant to all three drugs prescribed, while we found no evidence of HIVDR in the other two participants at 6 months (Id # 146 and 314) (Additional file 1: Table S1). The former had pre-existing mutation and acquired additional mutations developing high level resistance to all the three antiretroviral drugs during the course of treatment (Additional file 1: Table S1).

All cases of HIVDR involved at least one NNRTI mutation, with the K103N (3/9) being the most frequently observed. Combined NNRTI and NRTI mutations were seen in four of nine (44.4%) participants and involved M184V/I in all cases and K65R and D67G occurred in one participant in addition to the M184I mutation. A K65R mutation was also detected in a slow responding patient. While five of 11 participants with virological failure received ART regimens that were not or only partially active, the other four became resistant during the therapy in spite of a fully active ART regimens initiated at baseline (Additional file 1: Table S1).

A total of four participants, two from the virologicaly failing group and another two from the slow responders had no HIVDR mutations at 6 months. Self reported adherence data indicated that all participants with genotype data had good treatment adherence except two of the slow responders (Id # 301 and 314). Measurements of NNRTIs plasma concentrations indicated two out of nine participants with resistance mutations at 6 months (Id # 213 and 269) and one participant with no resistance mutation (Id # 357) had a drug concentration below the limit of quantification at 1 and 2 months (Table 2).

## Discussion

This report describes virological failure or slow virological response and associated HIVDR mutations in a cohort of HIV patients initiating first-line regimen in the Ethiopian national ART roll out program. The study revealed a virological failure rate of 5.3% and transmitted resistance among 6/12 of the virological failures in this cohort. Resistance mutations were detected in 9/11 (81.8%) of the patients failing treatment at 6 months. Although the virological failure rate was low, the transmitted resistance documented in this study, in a setting where ART has only been available for eight years is alarming.

A strength of this study is that patients were closely followed in a clinical trial unit for regular clinical and laboratory monitoring at 3 and 6 months, leading to reliable identification of virological failure and minimizing losses-to-follow-up. The potential limitation is that the patients participated in a nutritional intervention study in which they were motivated to come to the ART clinic and perhaps had better ART adherence, compared to patients accessing treatment in a regular setting. The drug resistance mutation analysis was done only on virological failures because of limited resource. Thus, the actual rate of transmitted drug resistance was not estimated.

Comparison of virological failure and HIVDR mutation rates obtained from different studies must be interpreted with caution, since threshold and duration of ART at time of failure varies. In a review from Sub-Saharan Africa countries (in which Ethiopia was not included), it was reported that virological success (defined by a VL < 400 copies/mL) rates after 6 months of ART was 78% [19]. In fact, 67% of the patients obtained viral suppression when applying lower cut-off values for success (VL less than 40 or 50 copies/mL). In this study, VL <40 copies/mL at 6 months was achieved in 87.9% of participants. Thus, compared with data from other Sub-Saharan African countries, the success rates found in the present study was higher using the same threshold.

The patterns of HIVDR mutations among the patients failing treatment in the present study differed from those described in previous two studies conducted in Ethiopia. In a study conducted in Gondar, only two mutations (V75I and G190A) were detected among 92 ART-naïve patients in 2003, before ART roll out [20]. Another investigation in Addis Ababa did not find any mutations among 39 women attending antenatal care units in 2005 [21]. In our study however, several mutations, which may cause moderate to high level of resistance to the NNRTIs and NRTIs were detected, indicating a changing pattern and a rise in transmitted resistance to antiretroviral drugs in Ethiopia.

Although derived from a small study, it is noticeable that 9/11 (81.8%) study participants genotyped at treatment failure had acquired or transmitted HIVDR, which is higher than the 63.7% reported from a study of 2000 HIV positive patients initiating first line ART using the WHO approach in other east African countries between 2006 and 2010 [22]. In three of nine participants with HIVDR mutations at baseline, exposure to a failing regimen during 6 months was associated with the emergence of additional mutations under drug pressure, including M184V/I. Among participants failing therapy and genotyped, two did not show any evidence of HIVDR mutations. Both participants reported very good adherence. However, efavirenz plasma concentrations in one of the participants were below limit of quantification at both 1 and 2 months visits. Although, reduced absorption could not be ruled out, the virological failure in this participant is likely due to undisclosed poor adherence [23].

Overall, the NRTI and NNRTI transmitted and acquired mutation patterns that were identified were consistent with previous reports in similar settings [19,24,25]. The most frequent NRTI mutation (M184V) and NNRTI mutations (K103N) described in this study are known to be common in cases of treatment failure [22]. The presence of transmitted resistance mutations has important implications for clinical management of HIV patients [26,27]. In addition, an immunological criterion alone has limitations to detect virological failures as shown in this study. These raise concerns about the routine use of the first-line regimens without access to viral load determination in Ethiopia.

Interestingly, one of the three patients exhibiting slow virological response had high level resistance to all the three drugs, although the VL was below 1000 copies/mL at 6 months. This may be due to the K65R mutation that can impair replicative fitness of the virus [28]. However, one of the virological failure also carried K65R; and the difference in virological outcome of these patients could be due to modulation of fitness cost by mutational interactions [29]. It is also interesting to note that many of the failing patients (70%) had VL >5000 copies/mL at 3 months in contrast to those with successful viral suppression, which highlights the potential of this criterion to identify virological failure and HIVDR earlier.

## Conclusions

In summary, we report virological success and resistance rates and pattern of HIVDR mutations among Ethiopian patients initiating first-line ART. After eight years of large-scale ART introduction, this survey demonstrated a low rate of virological failure. However, major mutations including K103N and M184V were identified, indicating that access to virological monitoring is of paramount importance to prevent inappropriate drug switches and preserve efficacy of ART in resource constrained countries. Moreover, the findings underscore the need for rapid and inexpensive tests to identify the most common drug resistant mutations.

## Abbreviations

ART: Antiretroviral therapy; HIVDR: HIV drug resistance; NNRTI: Non-nucleoside reverse transcriptase inhibitors; NNRTI: Nucleoside reverse transcriptase inhibitors; SSI: Statens Serum Institut; WHO: World Health Organization; VL: Viral load.

## Competing interests

The authors declare that they have no competing interests.

## Authors’ contributions

AAl, HF and ABA conceived the study and designed the study. AAl, DY, MFO, MT, PK and TG took care of participants’ recruitment. AAl was responsible for the VL measurements and drafted the manuscript. JF and LHC performed the HIVDR genotype analysis. AAl, ABA, CP, AMA and AAs contributed in the interpretation of results. All authors reviewed and approved the manuscript.

## Pre-publication history

The pre-publication history for this paper can be accessed here:

http://www.biomedcentral.com/1471-2334/14/181/prepub

## Supplementary Material

Additional file 1: Table S1 Known HIV drug resistance mutations and resistance in samples from patients with virological failure and slow virological responders at baseline and 6 months. Click here for file
