# Supplementary material for: Drug resistance in HIV patients with virological failure or slow virological response to antiretroviral therapy in Ethiopia
Source: BMC Infect Dis. 2014 Apr 4;14:181. doi: 10.1186/1471-2334-14-181 (PMC4234735; doi:10.1186/1471-2334-14-181)
Supplement: Additional file 1: Table S1 — Known HIV drug resistance mutations and resistance in samples from patients with virological failure and slow virological responders at baseline and 6 months. [file 1471-2334-14-181-S1.docx]

| Table S1. Known HIV drug resistance mutations and resistance in samples from patients with virological failure and slow virological responders at baseline and 6 months | | | | | | | | | | | | |
| --- | --- | --- | --- | --- | --- | --- | --- | --- | --- | --- | --- | --- |
| Patient Id | **Mutations and resistance at baseline** | | | | | **Mutations and resistance at 6 months** | | | | | **ART regimen and resistance at baseline ^1^** | **ART regimen and resistance at 6 months ^1^** |
|  | NNRTI | | NRTI | | | NNRTI | | NRTI | | |  |  |
|  | EFV | NVP | TDF | 3TC | AZT | EFV | NVP | TDF | 3TC | AZT |  |  |
| Virological failures | | | | | | | | | | | | |
| 9 | k103N, V90IV | |  |  |  | k103N, V90I, P225H | |  | M184I |  | TDF/3TC/**EFV** | TDF/**3TC/EFV** |
| 25 |  | A98G |  |  |  |  |  |  |  |  | TDF/3TC/EFV | TDF/3TC/EFV |
| 157 |  |  |  |  |  | G190Q, V90I | | M184I, K65KR, D67G | |  | TDF/3TC/EFV | **TDF/3TC/EFV** |
| 164 | NA | NA | NA | NA | NA | NA | NA | NA | NA | NA | AZT/3TC/NVP | AZT/3TC/NVP |
| 213 |  |  |  |  |  | Y181C | Y181C |  |  |  | TDF/3TC/NVP | TDF/3TC/**NVP** |
| 243 | K103KN | |  |  |  | K103KN | |  |  |  | AZT/3TC/**NVP** | AZT/3TC/**NVP** |
| 269 | V179D, V90I | |  |  |  | V179D, V90I | |  |  |  | TDF/3TC/**EFV** | TDF/3TC/**EFV** |
| 300 | K103N, V106MV | |  |  |  | K103N | |  |  |  | TDF/3TC/**EFV** | TDF/3TC/**EFV** |
| 339 | V90IV | |  |  |  | A98G  ,Y181C | A98G ,Y181C |  | M184I |  | TDF/3TC/NVP | TDF/**3TC/NVP** |
| 346 | K101E | K101E |  |  |  | K101E,G190A | |  | M184V |  | AZT/3TC**/NVP** | AZT/**3TC/NVP** |
| 354 |  |  |  |  |  | NA | NA | NA | NA | NA | TDF/3TC/EFV | TDF/3TC/EFV |
| 357 |  |  |  |  |  |  |  |  |  |  | TDF/3TC/EFV | TDF/3TC/EFV |
| 360 |  |  |  |  |  | V106M | |  |  |  | TDF/3TC/NVP | TDF/3TC/**NVP** |
| 373 | NA | NA | NA | NA | NA | NA | NA | NA | NA | NA | TDF/3TC/EFV | TDF/3TC/EFV |
| Slow responders | | | | | |  |  |  |  |  |  |  |
| 146 |  |  |  |  |  |  |  |  |  |  | TDF/3TC/EFV | TDF/3TC/EFV |
| 301 | V179DV | |  |  |  | V106M, V179D | | K65KR | K65KR |  | TDF/3TC/**EFV** | **TDF/3TC/EFV** |
| 314 |  |  |  |  |  |  |  |  |  |  | TDF/3TC/EFV | TDF/3TC/EFV |

NA: not applicable since samples were not adequate or failed to sequence.EFV=Efavirenz, NVP= Nevirapine, TDF=Tenofovir, 3TC=Lamivudine, AZT=Zidovudine. ^1^ Resistance is associated with reduced activity to the drugs in bold.

|  | Sensitive |
| --- | --- |
|  | Sensitive |
|  | Moderately resistant |
|  | Moderately resistant |
|  | Resistant |
